# The Role of ABA in Plant Immunity is Mediated through the PYR1 Receptor

**DOI:** 10.3390/ijms21165852

**Published:** 2020-08-14

**Authors:** Javier García-Andrade, Beatriz González, Miguel Gonzalez-Guzman, Pedro L. Rodriguez, Pablo Vera

**Affiliations:** Instituto de Biología Molecular y Celular de Plantas, Universidad Politécnica de Valencia-C.S.I.C, Ciudad Politécnica de la Innovación, Edificio 8E, 46000 Valencia, Spain; jagarsercs@gmail.com (J.G.-A.); beagong1@ibmcp.upv.es (B.G.); mguzman@uji.es (M.G.-G.); prodriguez@ibmcp.upv.es (P.L.R.)

**Keywords:** ABA, ethylene, pathogens, plant immunity, PYR1, salicylic acid

## Abstract

ABA is involved in plant responses to a broad range of pathogens and exhibits complex antagonistic and synergistic relationships with salicylic acid (SA) and ethylene (ET) signaling pathways, respectively. However, the specific receptor of ABA that triggers the positive and negative responses of ABA during immune responses remains unknown. Through a reverse genetic analysis, we identified that PYR1, a member of the family of PYR/PYL/RCAR ABA receptors, is transcriptionally upregulated and specifically perceives ABA during biotic stress, initiating downstream signaling mediated by ABA-activated SnRK2 protein kinases. This exerts a damping effect on SA-mediated signaling, required for resistance to biotrophic pathogens, and simultaneously a positive control over the resistance to necrotrophic pathogens controlled by ET. We demonstrated that PYR1-mediated signaling exerted control on a priori established hormonal cross-talk between SA and ET, thereby redirecting defense outputs. Defects in ABA/PYR1 signaling activated SA biosynthesis and sensitized plants for immune priming by poising SA-responsive genes for enhanced expression. As a trade-off effect, *pyr1*-mediated activation of the SA pathway blunted ET perception, which is pivotal for the activation of resistance towards fungal necrotrophs. The specific perception of ABA by PYR1 represented a regulatory node, modulating different outcomes in disease resistance.

## 1. Introduction

Pathogen recognition triggers the altered accumulation of three major defense hormones: salicylic acid (SA), jasmonic acid (JA), and ethylene (ET). SA is essential for establishing resistance to many virulent biotrophic pathogens, especially as a component of systemic acquired resistance (SAR) [1,2], while JA and ET tend to be associated with resistance to fungal necrotrophic pathogens [3,4]. While JA and ET interact synergistically to activate certain disease responses, the JA and ET pathways act at least independently or even antagonistically with respect to the SA-dependent pathway [4,5]. Antagonistic interactions between SA and JA hormone signaling networks have been characterized [6,7,8]. JA levels decline soon after SA begins to accumulate [9]; this, therefore, suggests that, in response to a pathogen that can induce synthesis of both SA and JA, cross-talk is used by the plant to adjust the response in favor of the more effective pathway (i.e., the SA-mediated pathway). Similarly, SA acts antagonistically with ET [10,11,12,13], and their biosynthesis pathways can be mutually repressed [14,15]. More recently, Huang et al. [16] revealed a mechanism by which SA antagonizes ET signaling: the direct interaction of NPR1 (the core component of SA signaling) with EIN3 (the transcription factor mediating ET-responses) blocks transcription of EIN3-induced genes, and this interaction is further enhanced by SA. Therefore, tradeoffs between plant defenses against pathogens with different lifestyles must be strictly regulated [4,17], implying the fine-tuned deployment of conserved defense signals in different plant-pathogen interactions.

ABA is another major phytohormone involved in the regulation of a great variety of abiotic stress responses in plants. In addition, ABA assists in controlling many developmental and growth characteristics of plants, including seed germination and dormancy, leaf abscission, closure of stomata, or inhibition of fruit ripening [18]. ABA also controls the responses of plants to biotic stresses caused by a broad range of plant pathogens [19,20,21,22]. However, the ABA effect varies in different pathosystems, being the outcome influenced by the infection biology. ABA biosynthesis is required for effective disease resistance against necrotrophic fungal pathogens [23,24,25], whereas ABA has been shown to be involved in conferring susceptibility against bacterial diseases, with ABA-deficient mutants showing resistance enhancement [21,26,27]. In fact, some bacteria have acquired new virulence strategies for exploiting their host through the secretion of type III virulence effectors that promote enhancement of ABA levels in the infected plant [28,29,30]. Therefore, endogenous ABA synergizes with JA and exhibits a complex antagonistic relationship with SA during disease development [6,7,29]. Likewise, antagonistic interactions between components of the ABA and ET signaling pathways seem to modulate gene expression in response to biotic and abiotic stress (Fujimoto et al., 2000; Chen et al., 2002; Anderson et al., 2004; Yang et al., 2005; Broekaert et al., 2006) [5,31,32,33,34], but it remains unknown whether a convergent point exists between these two signaling pathways or whether they operate in parallel. Despite all these evidences, the specific components of the ABA signaling apparatus, which exploit the positive and negative responses of ABA during immune responses, remain unknown. Therefore, understanding the regulatory system of ABA-mediated responses to pathogens is critical for improving agricultural issues related to disease resistance. In contrast, specific components of ABA perception have been recently identified for stomatal closure signal integration [35]. Thus, PYL2 is sufficient for guard-cell ABA-induced response, and PYL4/5 are essential receptors for a guard-cell response to CO_2_ [35].

Three major protein families form the core ABA signaling pathway; (i) the soluble ABA receptors, which are 14 members of pyrabactin resistance 1 (PYR1) and PYR1-like (PYL) proteins, also known as regulatory component of ABA receptors (RCAR) family and collectively referred to as PYR/PYL/RCAR, (ii) group A of type 2C protein phosphatases (PP2Cs), and (iii) SNF1-related protein kinases (SnRKs) subfamily 2 (SnRK2s), namely SnRK2.2, 2.3 and 2.6 (Cutler et al., 2010; Hubbard et al., 2010; Klingler et al., 2010; Raghavendra et al., 2010) [18,36,37,38]. In the absence of ABA, PP2Cs dephosphorylate and inactivate SnRK2s, repressing ABA-dependent responses [39,40]. When ABA concentration increases in response to stress conditions or developmental cues, ABA binds to receptors of the PYR/PYL/RCAR family, which leads to the formation of ternary complexes with PP2Cs, thereby inactivating them [41,42,43]. This results in the activation of SnRK2s, which subsequently phosphorylate a myriad of substrate proteins [44].

The PYR/PYL/RCAR ABA receptor family is unusually large, comprising 14 members in Arabidopsis and even more in crops, such as tomato, maize, or soybean (Gonzalez-Guzman et al., 2012; Gonzalez-Guzman et al., 2014; Helander et al., 2016) [45,46,47]. However, the biological roles of the individual PYR/PYL/RCAR members are still being established, which is complicated by functional redundancy. At least 13 PYR/PYL/RCAR members are able to perceive ABA, and the generation of quadruple, pentuple, and sextuple mutants is required to obtain robust ABA-insensitive phenotypes [41,43,45]. Moreover, the analysis of combined *pyr/pyl* mutants shows quantitative regulation of both stomatal aperture and transcriptional response to ABA [45]. Inactivation of six highly transcribed members, *PYR1*, *PYL1*, *PYL2*, *PYL4*, *PYL5*, and *PYL8*, generates a mutant that is practically blind to ABA in the classical assays that measure ABA sensitivity [45]. However, in spite of the receptor gene expression patterns and biochemical analyses of different receptor-phosphatase complexes suggesting that the function of ABA receptors is not completely redundant [45,48,49], only the single *pyl8* mutant has been reported to show a non-redundant role in root sensitivity to ABA [50]. In contrast, *pyr1* shows wild-type sensitivity to ABA and only shows a conditional phenotype -pyrabactin resistance in germination assays in medium supplemented with the ABA agonist pyrabactin [43]. In eukaryotes, functional diversification follows the evolutionary expansion of a gene family. Identification of specific roles for members of a multigene family is usually limited by laboratory conditions, whereas the plethora of conditions found in complex biological contexts offers chances to identify specific roles. Here, we were able to unveil a non-redundant role in plant immunity for PYR1, one of the 13 members of the multigene ABA receptor family, and revealed that the PYR1 receptor is pivotal in modulating the cross-talk between the SA and ET signaling pathways during the defense.

## 2. Results

### 2.1. The SnRK2s Protein Kinases are Engaged in Disease Resistance to Fungal Infection

**Figure 1:**
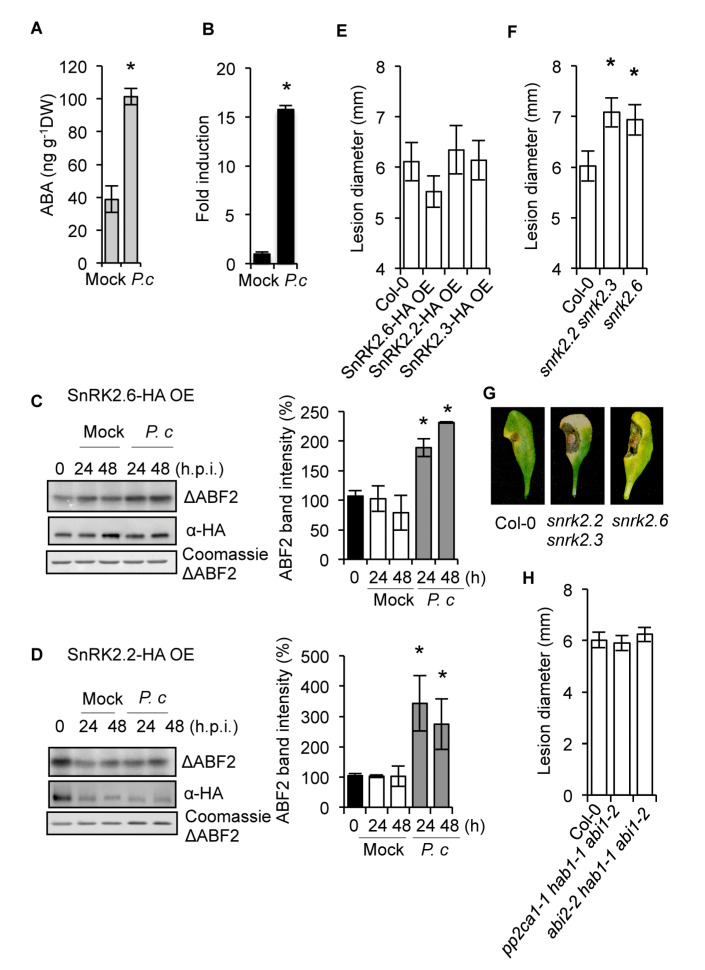
Participation of SnRK2s kinases in the response of Arabidopsis plants to infection by the fungal pathogen *P. cucumerina*. (**A**) ABA accumulation determined in mock and *P*. *cucumerina*-infected Col-0 plants. (**B**) RT-qPCR of *ABI4* in mock and in *P. cucumerina*-infected Col-0. (**C**,**D**) *P. cucumerina*-mediated activation of SnRK2.6 (**C**) and SnRK2.2 (**D**). Transgenic Arabidopsis plants expressing HA-tagged versions of the kinases were inoculated with *P. cucumerina*, or were mocked, and leaf samples were taken at 0, 24, and 48 h.p.i., and the protein extracts were immunoprecipitated with anti-HA antibodies. The immunoprecipitates were incubated with a His-ABF2 fragment (Gly73 to Gln 119; ΔABF2) in the presence of [γ-^32^P]ATP, and the proteins were resolved by SDS-PAGE. Bands corresponding to ΔABF2 fragments and to SnRK2.6 and SnRK2.2 kinases are indicated. Radioactivities of ΔABF2 fragment bands were measured with a phosphoimager, and the values were plotted on the graphs shown at the right of the figures. Error bars indicate S.E.M.; *n* = 3. (**E**) Disease resistance towards *P. cucumerina* of transgenic plants overexpressing SnRK2.6, SnRK2.2, and SnRK2.3 in comparison to Col-0. (**F**) Disease resistance towards *P. cucumerina* in the double *snrk2.2 snrk2.3* mutant and in *snrk2.6* mutant plants. (**G**) Representative leaves from each genotype at 12 days following inoculation with *P. cucumerina*. (**H**) Disease resistance towards *P. cucumerina* in the triple PP2C mutants *pp2ca1 1hab1 1abi1-2* and *abi2-2 hab1 abi1-2*. For the bioassays with *P. cucumerina*, lesion diameter of 25 plants per genotype and four leaves per plant were determined 12 d following inoculation with *P. cucumerina*. Data points represent the average lesion size ± SE of measurements. An ANOVA was conducted to assess significant differences in the activation of SnRKs, ABA accumulation, ABI4 transcript accumulation, and disease symptoms, with a priori *p* < 0.05 level of significance; the asterisks * above the bars indicate statistically significant differences regarding mock treatments or Col-0 plants. Asterisks above the bars indicate different homogeneous groups with statistically significant differences.

Liquid chromatography-mass spectrometry (LC-MS) showed marked accumulation of ABA in full expanded leaves of Arabidopsis plants at 72 h after drop inoculation with a spore suspension of the fungal necrotroph *Plectosphaerella cucumerina* (Figure 1A). ABA enhancement supported the upregulation of *ABI4* gene expression, an ABA-responsive gene encoding a transcription factor [23] (Figure 1B). Therefore, ABA biosynthesis and signaling were triggered by *P. cucumerina* infection. The ABA-mediated activation of three monomeric SnRK2s (i.e., SnRK2.2, −2.3, and −2.6) is central to ABA signaling [51], so we investigated whether SnRK2s were engaged in the defense responses to this pathogen. Transgenic lines overexpressing HA-tagged SnRK2.6 (SnRK2.6-HA/OE) and SnRK2.2 (SnRK2.2-HA/OE) were inoculated with *P. cucumerina* or mock-treated, and leaf samples were collected at 0, 24, and 48 h post-inoculation (h.p.i.). Immunoprecipitation of SnRK2.2-HA and SnRK2.6-HA and the subsequent kinase assay of the immunoprecipitate were performed by determining the incorporation of ^32^P to purified ABF2 protein fragment substrate (amino acids Gly-73 to Gln-119) [52] in gel-kinase assays. Results revealed two- and three-fold enhancement for SnRK2.6 and SnRK2.2 kinase activity, respectively, following fungal inoculation (Figure 1C,D). For both kinases, enhanced activity occurred at 24 h.p.i., and the activation was sustained at 48 h.p.i. Therefore, ABA-activated SnRK2s were actively engaged in response to this fungal pathogen.

**Figure 2:**
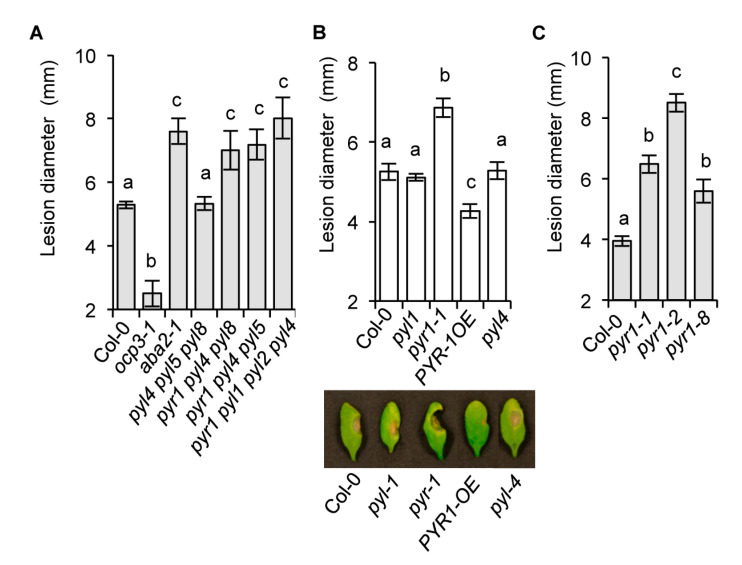
PYR1 is required for disease resistance towards *P. cucumerina*. (**A**). Disease resistance towards *P. cucumerina* in Col-0, the resistant *ocp3-1 mutant*, the susceptible *aba2-1*, and the triple and quadruple multi-locus mutants *pyl4 pyl5 pyl8*, *pyr1 pyl4 pyl8*, *pyr1 pyl4 pyl5*, and *pyr1 pyl1 pyl2 pyl4*. (**B**) Disease resistance in single *pyl1*, *pyr1*, and *pyl4* mutants, in a transgenic line overexpressing PYR1 (PYR1-OE), and in Col-0. Below the graph, the representative leaves from each genotype are shown at 12 days following inoculation with *P. cucumerina*. (**C**) Comparative disease resistance towards *P. cucumerina* among the allelic *pyr1-1*, *pyr1-2*, and *pyr1-8* mutants. Data points represent the average lesion size ± SE of measurements. An ANOVA was conducted to assess significant differences in disease symptoms (*p* < 0.05); the letters above the bars indicate different homogeneous groups with statistically significant differences.

We then investigated whether gain-of-function or loss-of-function in SnRK2s altered disease resistance to *P. cucumerina*. Symptoms of the fungal disease appear in the form of necrotic lesions, which are measured to quantify the degree of plant susceptibility [25,53,54]. Inoculation of transgenic plants individually overexpressing (OE) SnRK2.2, −2.3, and −2.6 revealed no significant variation in disease susceptibility towards *P. cucumerina* when compared to Col-0 plants (Figure 1E); thus, either endogenous SnRK2s levels are sufficient to achieve pathogen-triggered ABA signaling or overexpression of SnRK2s additionally requires increased ABA levels to enhance their activity. Although functional redundancy between SnRK2.2 and SnRK2.3 exists, functional segregation between SnRK2.6 and SnRK2.2/2.3 has been described [52]. Therefore, we inoculated an *snrk2.2/2.3* double mutant and the single *snrk2.6* mutant with *P. cucumerina* and recorded disease resistance. The triple *snrk2.2/2.3/2.6* mutant, which is drastically affected in plant growth [51], was not compatible with the pathogenic assay and was, therefore, not used in the present study. Figure 1F–G show that *snrk2.2/2.3* and *snrk2.6* plant resistance to *P. cucumerina* was severely compromised. Moreover, an ABA deficient mutant (i.e., *aba2*) was similarly affected in disease resistance to this pathogen (Figure 2A). In summary, our results indicated that pathogen-induced ABA accumulation and concurrent activation of SnRK2s positively regulated disease resistance to *P. cucumerina*.

ABA signaling through SnRK2s is negatively regulated by clade A protein phosphatase type 2C (PP2C), particularly by ABI1, ABI2, PP2CA/AHG3, AHG1, HAB1, and HAB2 (see [55] and references therein). Therefore, clade A PP2Cs might negatively regulate ABA-mediated disease resistance to *P. cucumerina*. Because of the demonstrated redundancy existing for these PP2Cs, combined inactivation of selected groups of these phosphatases is required to determine functionality. We combined loss-of-function mutations in ABI1, ABI2, HAB1, and PP2CA genes to determine their contribution to ABA-mediated disease resistance. Different combinations of mutations were used with two triple mutants, *pp2ca1-1;hab1-1;abi1-2* and *abi2-2;hab1-1;abi1-2*, which represent four of the nine closely related group A PP2Cs. Both multi-locus mutants showed an extreme response to exogenous ABA, partial constitutive response to endogenous ABA, and partial constitutive activation of SnRK2s in *pp2ca1-1;hab1-1;abi1-2* [51,55]. Inoculation of both triple mutants with *P. cucumerina* showed no defective disease resistance (Figure 1H). This result suggests that the demonstrated redundancy of PP2Cs masks the manifestation of a clear phenotype upon pathogen inoculation. Additionally, ABA response in triple *pp2c* mutants was partially equivalent to that of lines OE SnRK2s, which did not show altered disease resistance to the pathogen (Figure 1E). It is also possible that other members of the large PP2C family, represented by 76 homologous genes [56], are key for resistance to *P. cucumerina*. This interpretation is supported by previous studies showing that a distinct PP2C member (i.e., AtDBP1) is required for other aspects of plant immunity [57,58]. 

### 2.2. The Requirement of the PYR1 Receptor for Antifungal Resistance

We next investigated which one of 14 soluble PYR/PYL/RCAR receptors perceived the ABA produced during *P. cucumerina* infection. Partial functional redundancy of ABA receptors has been demonstrated by genetic analysis; however, PYL8 plays a non-redundant role to regulate root sensitivity to ABA [45,46]. Additionally, both transcriptional and physiological ABA responses and signaling of environmental cues in guard cells mediated by individual receptors are starting to be elucidated [35]. We characterized disease resistance to *P. cucumerina* in a series of multi-locus mutants from different PYR/PYL receptors. The triple *pyl4;pyl5;pyl8*, *pyr1;pyl4;pyl8,* and *pyr1;pyl4;pyl5* mutants, and the quadruple *pyr1;pyl1;pyl2;pyl4* mutant, representing the highest genetic impairment in PYR/PYL function without affecting plant growth [45], were inoculated with *P. cucumerina*, and their impact on disease resistance was compared to *aba2-1* (which enhances susceptibility [25]), to *ocp3-1* (which enhances resistance [53]), and Col-0 plants. The two triple mutants incorporating the *pyr1* mutation (i.e., *pyr1;pyl4;pyl8* and *pyr1;pyl4;pyl5*) exhibited noticeably enhanced disease susceptibility (Figure 2A), which was of a magnitude similar to that observed in *aba2-1* plants. Conversely, the disease resistance of the triple *pyl4;pyl5;pyl8* mutant was unaltered compared to Col-0 plants. The quadruple mutant (also containing the *pyr1* mutation) enhanced disease susceptibility to *P. cucumerina*. The results showed that the PYR1 receptor was pivotal for eliciting ABA-mediated defense responses towards *P. cucumerina*. 

The specificity of PYR1 at eliciting plant immune responses was further tested by assaying the single *pyr1-1* mutant. The individual *pyr1-1* mutant had a compromised disease resistance phenotype (Figure 2B), contrasting to other single *pyl* mutants (e.g., *pyl1*, *pyl4*) for which resistance to the fungus remained intact. Moreover, the overexpression of the PYR1 receptor (PYR1-OE line) conferred significant enhancement of resistance to the fungus (Figure 2B). Other mutant alleles of the PYR1 receptor, predicted to produce a variety of defects in PYR1 (i.e., *pyr1-2* and *pyr1-8* [43]), consistently compromised disease resistance to *P. cucumerina*, showing *pyr1-2* mutant allele as the strongest phenotype (Figure 2C). These results supported that the PYR1 receptor positively promoted ABA-dependent plant immunity against *P. cucumerina*. Interestingly, these results also indicated that other major receptors for ABA response, i.e., PYL1, PYL4, PYL5, PYL8, were not recruited in plant response against *P. cucumerina*. Furthermore, PYR1 appeared similarly to be required for the immune activation to *Alternaria brassicicola*, another fungal necrotroph and the causal agent of black spot disease in Brassica species. Results shown in Supplemental Figure S1 indicate that upon inoculation with *A. brassicicola*, both *aba2* and *pyr1* plants, compared to Col-0, *pyl1*, and *pyl4* plants, showed remarkable enhancement in disease susceptibility to this pathogen. The enhancement of necrosis in *A. brassicicola*-inoculated leaves of *pyr1* plants gave further support to the importance of PYR1-mediated perception of ABA for mounting effective defense responses towards necrotrophs.

### 2.3. Local Induction of PYR1 Gene Expression by P. cucumerina

**Figure 3:**
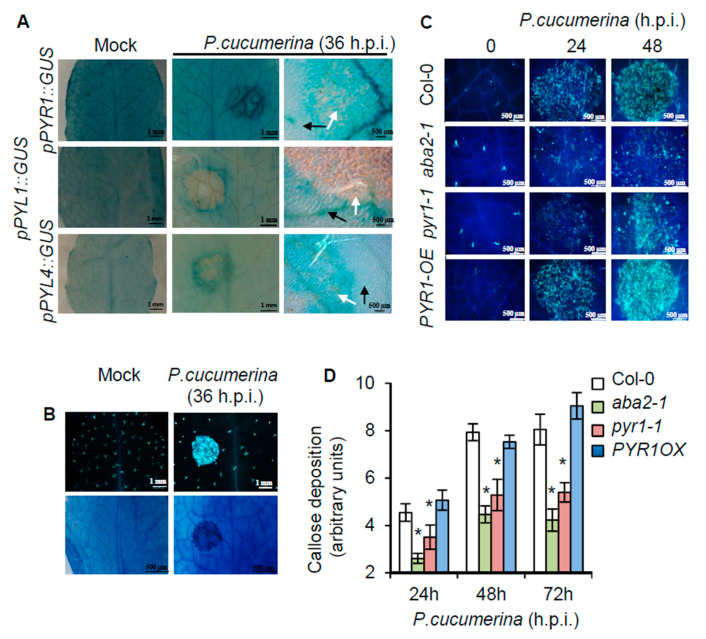
Local activation of *PYR1* gene expression at pathogen inoculation sites, and the requirement of PYR1 for pathogen-induced callose deposition. (**A**) Comparative histochemical analysis of GUS activity in rosette leaves from transgenic plants carrying *pPYR1::GUS*, *pPYL1::GUS*, and *pPYL4::GUS* gene constructs and those were either mocked or inoculated *P. cucumerina*. Leaves were stained for GUS activity at 36 h.p.i. The left panel corresponds to mocked plants. The central and right panels correspond to enlargements of the inoculated leaf sectors. Black arrow points towards leaf tissues proximal to the inoculation point, and white arrows denote tissues that directly received the spore inoculum. Note that *pPYR1::GUS* is heavily induced in leaf veins within the inoculated sector. (**B**) Characteristic spore-inoculated leaf sector, similar to those shown in A, stained with aniline blue to detect pathogen-induced callose deposition (top panel), or with trypan blue (lower panel) to identify incipient cell deterioration due to fungal infection at 36 h.p.i. (**C**) Aniline blue staining and epifluorescence microscopy were applied to visualize callose accumulation. Micrographs indicate *P. cucumerina* inoculation and infection site in the different Arabidopsis genotypes at 0 h.p.i (right panel), at 24 h.p.i. (central panel), and at 48 h.p.i. (right panel). (**D**) The number of yellows pixels (corresponding to pathogen-induced callose) per million on digital photographs of infected leaves were used as a means to express arbitrary units (i.e., to quantify the image) at the indicated times. Bars represent mean ± SD, *n* = 15 independent replicates. An ANOVA was conducted to assess significant differences in callose deposition (*p* < 0.05); the asterisks * above the bars indicate statistically significant differences regarding Col-0 plants.

A reasonable explanation for the specific role of PYR1 in plant immunity might be the specific upregulation of *PYR1* expression in response to the pathogen. Therefore, we next investigated whether transcriptional reprogramming occurred to enhance *PYR1* expression upon pathogen inoculation. Transgenic plants expressing the promoter of the *PYR1* gene fused to the β-glucuronidase GUS reporter gene (*pPYR1::GUS*) [45] were used to detect potential *P. cucumerina*-mediated activation of *PYR1*. Transgenic lines carrying the *pPYL1::GUS* and *pPYL4::GUS* gene constructs were also assayed to determine specificity. Local infection, i.e., by drop inoculation on the upper leaf surface with a *P. cucumerina* spore suspension, of transgenic *pPYR1::GUS* plants revealed early transcriptional activation of *PYR1* triggered by the pathogen (Figure 3A). *PYR1* induction mostly occurred within the vascular bundles of the primary and secondary veins of the *P. cucumerina*-inoculated leaf sectors. This highly localized induced expression pattern was specific to *PYR1* because neither *PYL1* nor *PYL4* genes were transcriptionally activated under similar circumstances (Figure 3A). The local induction of *pPYR1::GUS* concurred with local synthesis and deposition of callose (Figure 3B) and later on with cell death (Figure 3C). These microscopy markers demarcated inoculated tissue sectors in advance to the appearance of visible necrosis and served to delimit local transcriptional responses. Moreover, callose deposition was compromised in *pyr1-1* and *aba2-1* mutants following fungal infection (Figure 3D), thus supporting the participation of ABA and PYR1 in this local process.

### 2.4. Resistance Enhancement of pyr1 Plants to Pseudomonas syringae DC3000

**Figure 4:**
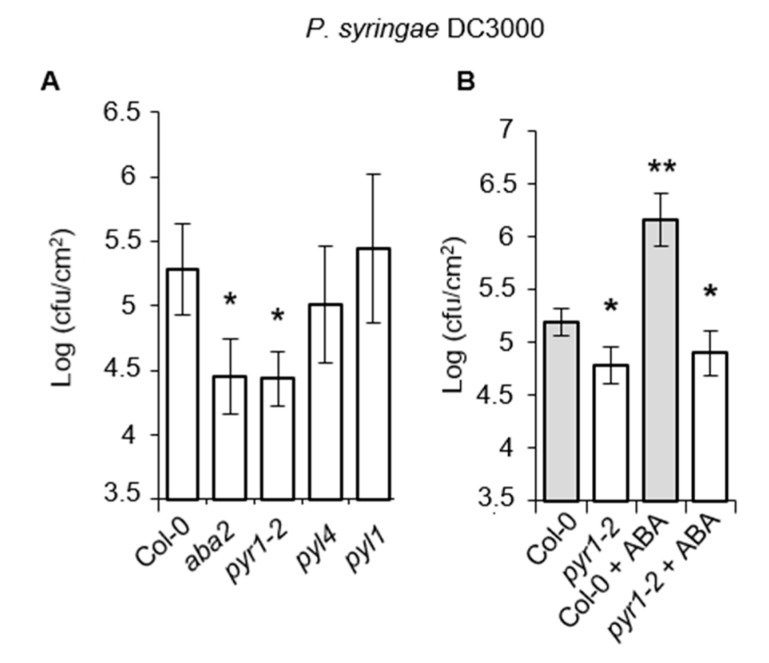
Response of *pyr1* plants to infection by *P. syringae DC3000*. (**A**) Col-0, *aba2-1*, *pyr1-2*, *pyl1*, and *pyl4* mutants were inoculated with *P. syringae* DC3000, and their disease responses were recorded. (**B**) Col-0 and *pyr1* plants were pre-treated with 150 μM ABA, applied by drenching, before inoculation with *P. syringae* DC3000, and the growth of the bacteria was recorded in comparison to mocked plants. Growth of *P. syringae* DC3000 was measured at 3 d.p.i. Error bars represent standard deviation (*n* = 12). An ANOVA was conducted to assess significant differences in disease symptoms, with a priori *p* < 0.05 level of significance; the asterisks *, ** above the bars indicate different homogeneous groups with statistically significant differences.

In marked contrast to the results shown above, the role of ABA in repressing plant immunity against the (hemi) biotrophic pathogens *P. syringae* DC3000 has been previously documented [6,19,20,21]. Therefore, we asked whether the negative role of ABA in plant immunity against *P. syringae* DC3000 could similarly be funneled through PYR1. If so, we would expect resistance enhancement in *pyr1* plants. *pyr1* plants were inoculated by leaf infiltration with *P. syringae* DC3000, and the rate of bacterial growth in the inoculated leaves was determined at 3 days post-inoculation in comparison to *aba2*, *pyl1*, *pyl4*, and Col-0 plants. Figure 4A shows that bacterial growth was reduced 10-fold in both *aba2* and *pyr1* mutants compared to Col-0, *pyl1*, and *pyl4*. This result confirmed the negative role of ABA in resistance towards *P. syringae* DC3000 and demonstrated the specific requirement of PYR1 for the negative role of ABA during this plant-pathogen interaction. Moreover, pre-treatment of Col-0 with 150 μM ABA, applied by drenching, predictably provoked disease susceptibility enhancement to *P. syringae* DC3000 (Figure 4B), denoting a damping effect of ABA on SA signaling. This ABA-mediated enhancement in susceptibility to *P. syringae* DC3000 did not occur in *pyr1-2* plants whose enhanced resistance was not altered by the hormone (Figure 4B).

Therefore, our results indicated that the dual antagonistic role of ABA in plant immunity was mediated through the PYR1 receptor, which reciprocally activates and represses immune responses towards necrotrophic and biotrophic pathogens, respectively.

### 2.5. SA–Responsive Defense Genes are Activated in PYR1 Defective Mutants

**Figure 5:**
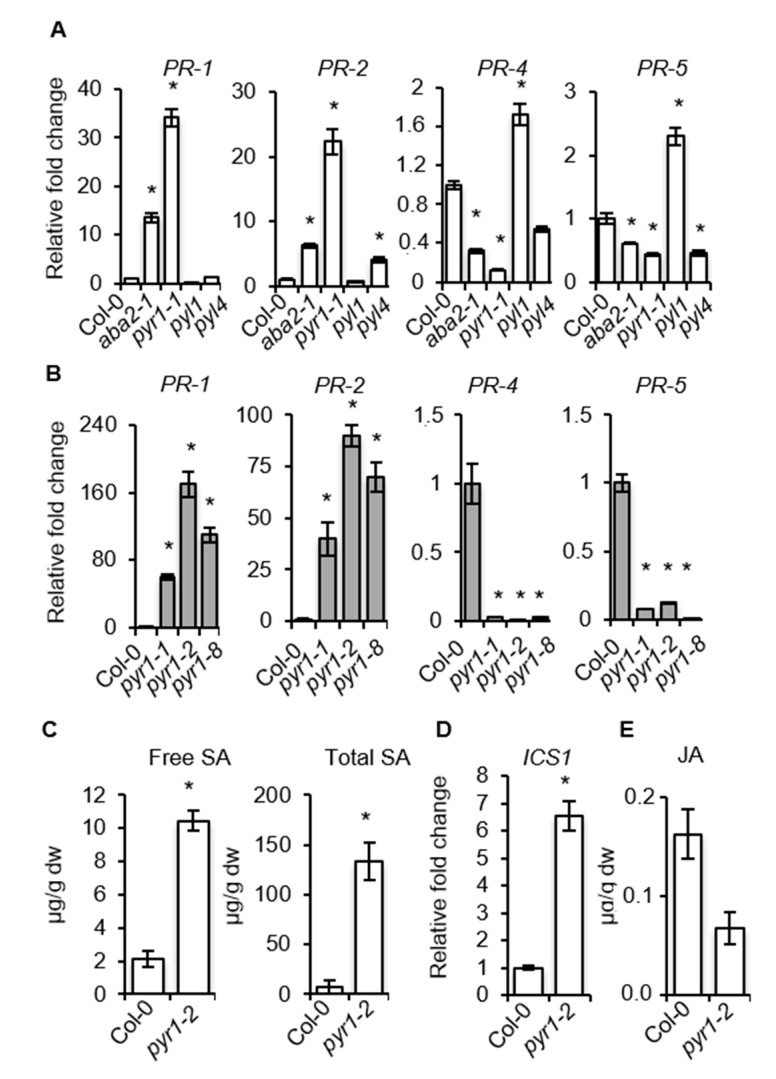
Expression of SA-responsive and ET-responsive genes in *pyr1* and *aba2* mutants. (**A**,**B**) RT-qPCR analysis showing constitutive expression levels of *PR-1*, *PR-2*, *PR-4*, and *PR-5* genes in (**A**) Col-0, *aba2-1*, *pyr1-1*, *pyl1*, and *pyl4* plants, and (**B**) their comparative expression levels in the allelic *pyr1-1*, *pyr1-2*, and *pyr1-8* mutants. Data represent mean ± SD; *n* = 3 replicates. The expression was normalized to the constitutive *ACT2* and *ACT8* genes and then to the expression in Col-0 plants. (**C**–**E**) Accumulation of free SA, total SA, and total JA in Col-0 and *pyr1-2* plants. Data represent the average of three biological replicates. An ANOVA was conducted to assess significant differences in RT-qPCR and hormone analysis, with a priori *p* < 0.05 level of significance; the asterisks * above the bars indicate statistically significant differences regarding Col-0 plants.

We investigated whether *pyr1* and *aba2* plants carried constitutive elevated expression of SA-responsive genes, which might explain the observed enhanced resistance to *P. syringae* DC3000 (Figure 4). The accumulation of *PR-1* and *PR-2* transcript, which are SA- and pathogen-responsive genes, was examined by RT-qPCR. In addition, we examined *PR-4* and *PR-5*, which are also pathogen-responsive genes but are simultaneously influenced by SA and ET [59]. Transcript accumulation was also evaluated in *pyl1* and *pyl4* mutants, which served as additional controls. Figure 5A shows that *pyr1* and *aba2* plants carried constitutive elevated levels of SA-dependent *PR-1* and *PR-2* transcripts compared to Col-0, *pyl1*, or *pyl4* plants. Conversely, the constitutive levels of transcript accumulation for *PR-4* and *PR-5* occurring in Col-0 were repressed in *pyr1* and *aba2* plants, and only partially enhanced in *pyl1* plants (Figure 5A). The enhanced expression of *PR-1* and *PR-2* and the concerted repression of *PR-4* and *PR-5* were corroborated by the *pyr1* allelic series, with the *pyr1-2* allele showing the strongest differences (Figure 5B). Thus, the ABA/PYR1 module might function as an integration node regulating distinct branches of defenses. The constitutive activation of *PR1*- and *-2* in *pyr1* plants supported the enhanced accumulation of both free and conjugated SA observed in the mutant, which concurred also with elevated expression of *ICS1*, encoding isochorismate synthase, a pivotal enzyme controlling SA biosynthesis [60,61] (Figure 5C,D). On the other hand, *pyr1* plants only showed a moderate reduction, less than two-fold, of JA content in comparison to Col-0 (Figure 5E). The conspicuous enhancement in SA content in healthy *pyr1* plants, therefore, explained the resistance phenotypes of the mutant when confronted with *P. syringae* DC3000. However, the notorious enhanced susceptibility of *pyr1* plants to the fungal necrotrophs *P. cucumerina* and *A. brassicicola* remained unsolved, as it could not simply be explained by the moderate reduction of JA levels as attained in the mutant.

### 2.6. Enhanced Activation of MAPK Kinases in pyr1 Plants

**Figure 6:**
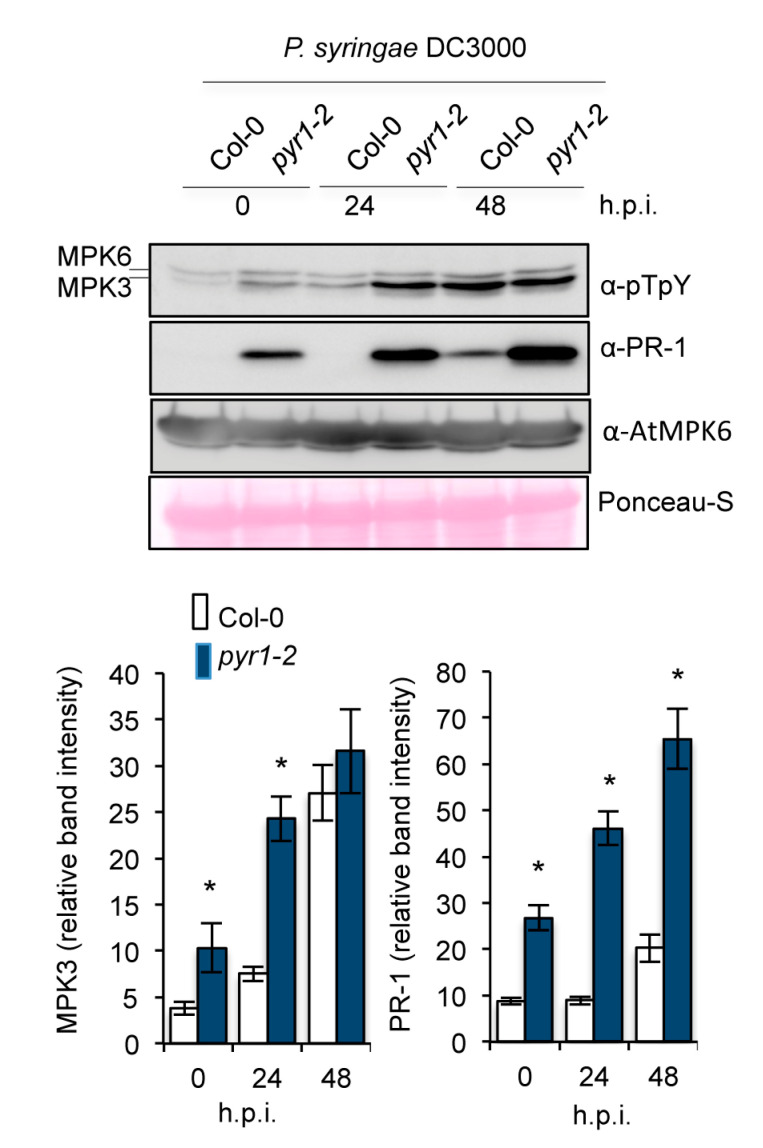
Loss of PYR1 function confers enhanced mitogen-activated kinase activation and PR-1 protein accumulation following *P. syringae* DC3000 infection. Western blot with anti-pTEpY and anti-PR-1 antibodies of crude protein extracts derived from Col-0, *pyr1-2* plants at 0, 24, and 48 h.p.i with *P. syringae* DC3000. Equal protein loading was check by Ponceau-S staining of the nitrocellulose filter. MPK6 and MPK3 migrating bands are indicated on the right. The experiments were repeated three times with similar results. Scan quantification of protein bands corresponding to MPK3 and PR-1 is shown below the Western blot. Data represent the mean ± SD; *n* = 3 replicates. An ANOVA was conducted to assess significant differences in RT-qPCR analysis, with a priori *p* < 0.05 level of significance; the asterisks * above the bars indicate statistically significant differences regarding Col-0 plants.

We next investigated whether enhanced resistance to *P. syringae* DC3000 in *pyr1* plants was associated with elevated MAPKs activation, which is linked to the activation of immune responses following pathogen perception. We employed an antibody recognizing the phosphorylated residues within the MAPK activation loop (i.e., the pTEpY motif). Western blot analysis of protein extracts derived from healthy Col-0 and *pyr1* plants showed positive immunoreactive signals in two polypeptides corresponding to MPK6 and MPK3 (Beckers et al., 2009) (Figure 6), and the densitometric scanning of blots indicated that the MPK3 immunoreactive band was more intense in *pyr1* plants. Inoculation with *P. syringae* DC3000 promoted further activation-associated dual TEY phosphorylation of MPKs, which was noticeably higher for MPK3 in *pyr1* compared to Col-0 plants at 24 h.p.i. (Figure 6). At the latter stages of infection (i.e., 48 h.p.i.), the MPK activation was similar in Col-0 and *pyr1* plants. Therefore, MPK activation may be prone to activation in plants defective of ABA perception through the PYR1 receptor. Indeed, partial pre-activation of MPK was reflected in detectable PR-1 protein accumulating in *pyr1* plants at time zero (Figure 6). This result was in agreement with the higher expression level of the *PR-1* gene determined by RT-qPCR (Figure 5A,B). Interestingly, inoculation with *P. syringae* DC3000 promoted the further accumulation of the PR-1 protein, which progressively increased over time to a much higher level in the *pyr1* mutant compared to Col-0 plants (Figure 6).

Thus, we hypothesize that the lack of ABA perception through the PYR1 receptor de-represses a pathway that allows cell sensitization through MPKs activation and downstream defense gene reprogramming, even in the absence of pathogen infection. Sensitized cells may be ready for the enhanced induction of this defense pathway following pathogen infection, which, in turn, may explain why *aba* and *pyr1* plants exhibit enhanced disease resistance to *P. syringae* DC3000. These observations support that ABA and PYR1 function as a repressor module of SA-mediated onset of resistance.

### 2.7. SA-Mediated Defense Genes are Poised for Enhanced Activation through Chromatin Remodeling in pyr1 Plants

**Figure 7:**
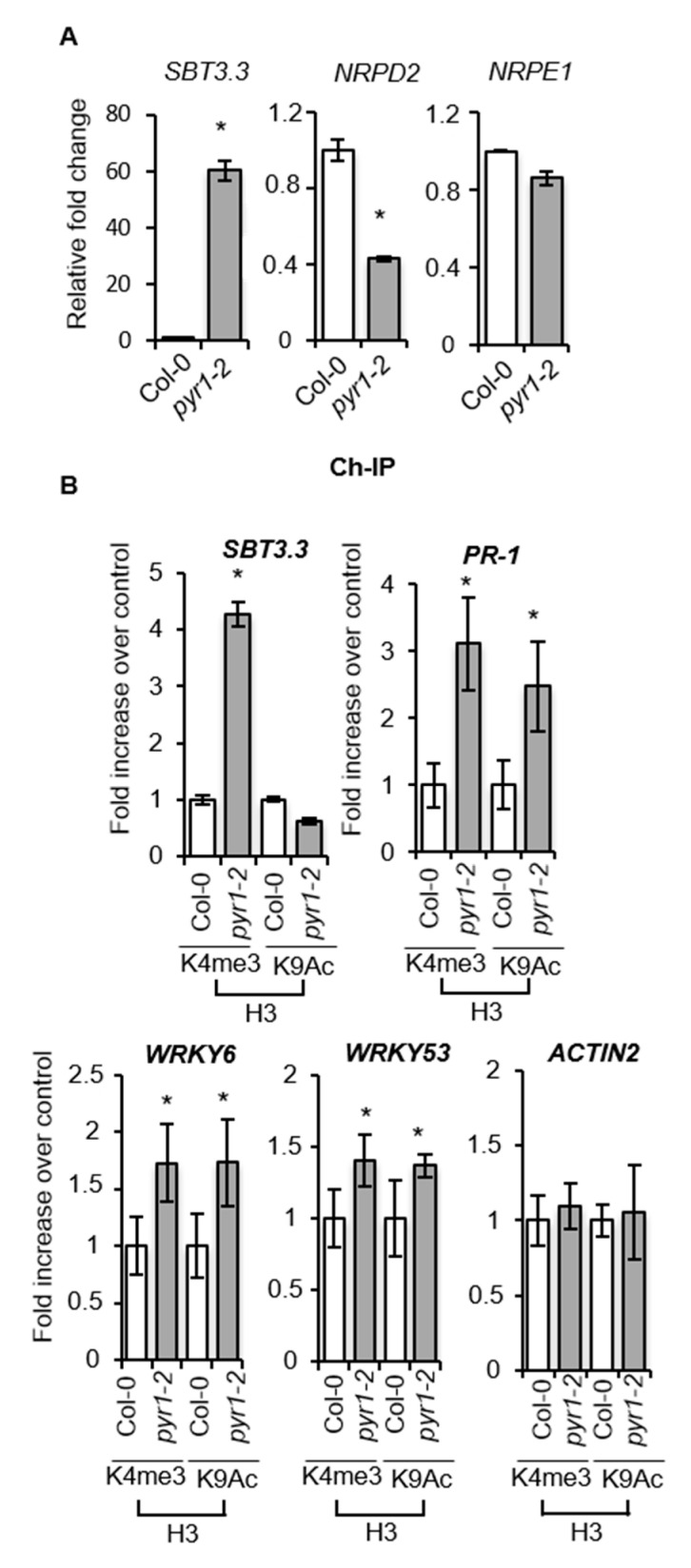
Loss of PYR1 function provokes the setting of hallmarks characteristic of primed immunity. (**A**) Comparative RT-qPCR of *SBT3.3*, *NRPD2*, and *NRPE1* transcript levels between healthy Col-0 and *pyr1-2* plants. The expression was normalized to the constitutive *ACT2/8* gene and then to the expression in Col-0 plants. (**B**) Chromatin immunoprecipitation (ChIP) and comparison between Col-0 and *pyr1-2* plants of the level of histone H3 Lys4 trimethylation (H3K4me3) and histone H3 Lys9 acetylation (H3K9ac) on the *SBT3.3*, *PR-1*, *WRKY6*, and *WRKY53* gene promoters as present in leaf samples. The setting of histone marks in *ACTIN2* was used as an internal control. Data are standardized for Col-0 histone modification levels. Data represent the mean ± SD; *n* = 3 biological replicates. An ANOVA was conducted to assess significant differences between MPKs activation and PR1 accumulation (*p* < 0.05); the asterisks * above the bars indicate statistically significant differences regarding Col-0 plants.

We then asked whether other markers diagnostic of an immune status were also activated in *pyr1* plants. The expression of the extracellular subtilase *SBT3.3* gene has been recently described to be a switch for poising SA-related gene expression and immune priming [62]. Moreover, constitutive *SBT3.3* expression, MPK activation, and readied SA-related genes convey in plants defective in the RNA-directed DNA methylation (RdDM) pathway, which negatively regulates immune priming [54]. Consequently, the expression level of genes encoding SBT3.3 and either of the two subunits of RNA Pol V (i.e., NRPD2 and NRPE1) controlling RdDM were determined by RT-qPCR. Figure 7A shows the constitutive upregulation of *SBT3.3* and concurrent downregulation of *NRPD2* in *pyr1* plants compared to Col-0, congruent with the activation of immune priming in the mutant. The downregulation was specific for *NRPD2*, encoding the second large subunit of Pol V, because the expression of the gene encoding the large NRPE1 subunit exhibited a minimal variation in *pyr1* plants (Figure 7A).

In plants defective in RdDM-mediated epigenetic control, immune priming is activated concurrently with chromatin histone activation marks being enriched in SA-related genes, including the *SBT3.3* gene itself [54,62]. Thus, we hypothesized that SA-related defense genes and *SBT3.3* in *pyr1-2* plants are poised for enhanced expression by differential histone modification. We used chromatin immunoprecipitation (ChIP) to analyze H3K4me3 and H3K9ac activation marks on the *SBT3.3* and *PR-1* gene promoter regions in *pyr1* and Col-0 plants. We also examined the genes encoding WRKY6 and WRKY53, transcriptional regulators of SA-defense genes. Figure 7B shows that H3K4me3 marks in the *SBT3.3* promoter region notably increased in *pyr1* plants compared to Col-0 plants, while H3K9ac marks remained invariant (Figure 7B), supporting previous descriptions of plants constitutively expressing primed immunity [54,62,63]. On the *PR1* promoter, both H3K4me3 and H3K9ac activation marks increased three- and two-fold, respectively, in *pyr1* plants compared to Col-0 (Figure 7B). Likewise, histone activation marks also moderately increased in the *WRKY6* and *WRKY53* promoters of *pyr1* plants compared to Col-0 plants (Figure 7B). The setting of histone marks in *pyr1* plants remained unchanged in the *ACTIN2* gene promoter, which was used as the control (Figure 7B). Therefore, chromatin activation marks proliferated in the promoter regions of the priming regulatory gene *SBT3.3* and the SA-responsive genes in *pyr1* plants and would explain why the PR-1 protein showed accelerated and enhanced accumulation in *pyr1* plants following pathogen inoculation (Figure 6). Our results indicated that ABA and its PYR1-mediated perception represented novel integral components of a signaling process, repressing SA-mediated immunity.

### 2.8. NahG Plants Abrogate the Altered Disease Resistance Response of pyr1 Plants

**Figure 8:**
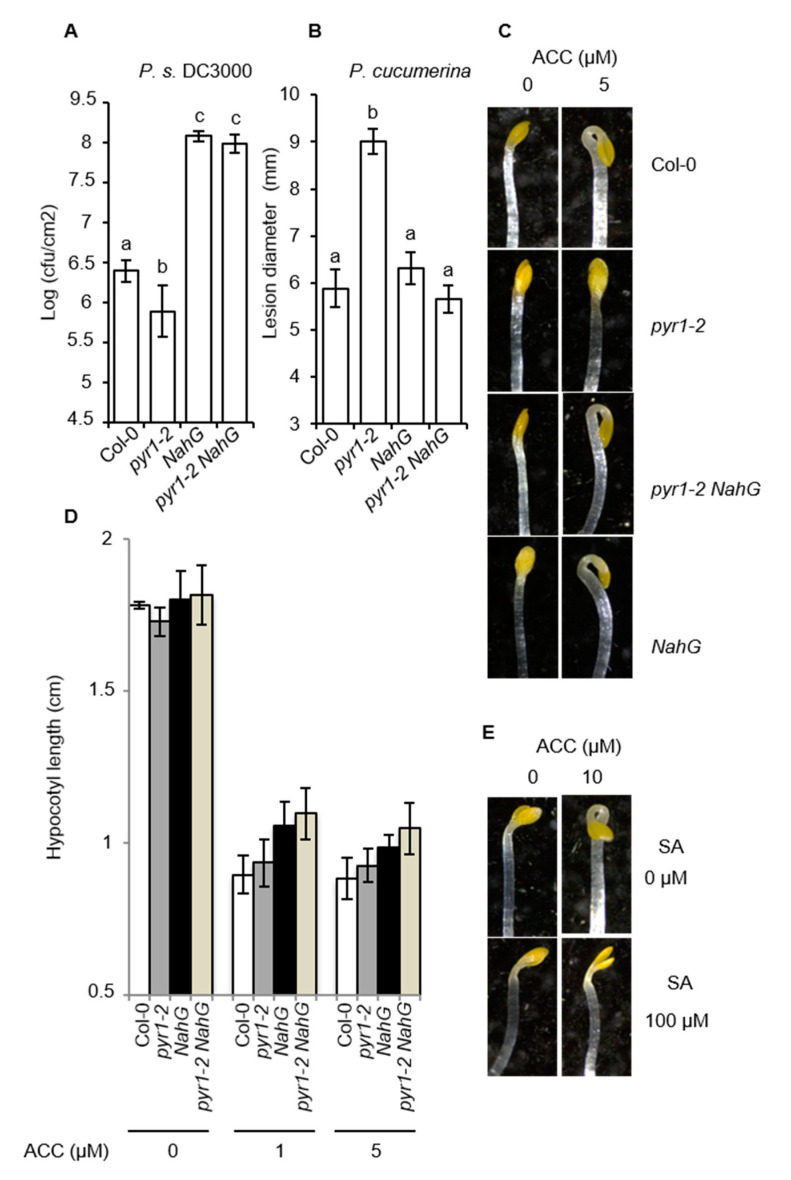
Effect of *NahG* on disease resistance and insensitivity to ACC of *pyr1* plants and seedlings. (**A**,**B**) Comparative disease resistance towards *P.s.* DC3000 and *P. cucumerina* among Col-0, *pyr1-2*, *NahG*, and *pyr1-2NahG* plants. Growth of *P. syringae* DC3000 was measured at 3 d.p.i. Error bars represent standard deviation (*n* = 12). For *P. cucumerina*, data points represent the average lesion size ± SE of measurements. An ANOVA was conducted to assess significant differences in disease symptoms (*p* < 0.05); the letters above the bars indicate different homogeneous groups with statistically significant differences. (**C**) Apical hook region of the indicated seedlings germinated and grown on MS/2 in the dark for 4 d in the presence of the indicated concentration of ACC. (**D**) Hypocotyl length of seedlings germinated and grown in the dark for 4 d on MS/2 medium supplemented with the denoted concentrations of ACC. Error bars represent standard deviation (*n* = 50). An ANOVA was conducted, and no significant differences were observed in hypocotyl length (*p* < 0.05). (**E**) Apical hook region of Col-0 seedlings germinated and grown on MS/2 in the dark for 4 d in the presence of the indicated concentration of ACC and SA.

To evaluate the role of SA for *pyr1*-altered resistance, we generated a *pyr1;NahG* double mutant. In plants carrying the *NahG* transgene, salicylate hydroxylase depletes the plant of this defense hormone [64]. Compared to Col-0, *NahG* plants showed an anticipated increase in susceptibility to *P. syringae* DC3000 due to SA depletion (Figure 8A). Interestingly, in *pyr1;NahG* plants, the *pyr1*-mediated enhanced resistance was abrogated, and instead enhanced susceptibility to *P. syringae* DC3000 emerged (Figure 8A). Moreover, when assayed against the fungal pathogen *P. cucumerina*, *NahG* plants behaved like Col-0, both showing the same degree of susceptibility (Figure 8B), suggesting normal metabolic levels of SA played no major role in the resistance towards this pathogen. Surprisingly, in *pyr1;NahG* plants, the *pyr1*-mediated-enhanced susceptibility to *P. cucumerina* was abrogated (Figure 8B), with *pyr1;NahG* plants to be behaving as Col-0 or *NahG* plants. This suggested that the PYR1-mediated perception of ABA negatively regulated the SA pathway. When this negative regulation failed, such as in *pyr1* plants, the SA levels increased, and the resistance to *P. syringae* DC3000 was activated. As a trade-off effect, the elevated SA levels presumably interfered with JA or ET signaling pathways required for mounting a resistance response to fungal pathogens.

### 2.9. pyr1-Mediated Enhanced SA Content Blocks ET Perception

The SA and JA signal pathways are under an antagonistic equilibrium. Therefore, we wondered if the enhanced SA levels of *pyr1* plants could be affecting JA signaling in this mutant. We studied *pyr1* plants for altered responses to JA using the widely applied root growth inhibition assay. In the absence of JA, primary root length of *pyr1* seedlings was comparable to that of Col-0 plants (Supplemental Figure S2), and in the presence of JA, root growth reduction in the mutant was also similar to that observed in Col-0 plants (Supplemental Figure S2), providing evidence that JA perception was not impaired in the mutant. In addition, comparison of the expression level of different JA-responsive genes at different times following *P. cucumerina* inoculation in Col-0 and *pyr1* plants revealed that JA signaling appeared to be not affected in the mutant (Supplemental Figure S3). Instead, for some of the genes analyzed, a higher induction was recorded in *pyr1* plants. Therefore, JA signaling was not compromised in *pyr1* plants.

We next asked whether ET signaling, which is also pivotal for resistance to fungal pathogens [10,12,13], could be the one impaired in *pyr1* plants due to the elevated levels of SA. This hypothesis gained even more relevance in view of the recently described mechanism explaining the antagonism between SA and ET in the suppression of apical hook formation and early seedling establishment via NPR1-mediated repression of EIN3 and EIL1 [16]. We, therefore, assayed Col-0 and *pyr1* seedlings, grown in the dark in the presence or absence of a low concentration of the ethylene precursor ACC (5 μM), for the induction of the ET-mediated triple response. The triple response in Arabidopsis consists of shortening and thickening of hypocotyls and roots and exaggeration of the curvature of apical hooks. Compared to Col-0, the assay revealed that *pyr1* seedlings showed no curvature of the apical hook (Figure 8C) and also showed less shortened hypocotyls (Figure 8D) when grown in the presence of ACC. Thus, the *pyr1* mutant was impaired in ET perception. The enhanced SA content in *pyr1* seedlings was the causal link mediating insensitivity to ET since in *pyr1;NahG* double mutant, normal sensitivity to ET was re-established (Figure 8C,D). Moreover, when Col-0 seedlings were assayed in the presence of high amounts of SA (100 μM), the ACC-induced triple response was abrogated (Figure 8E), further sustaining that the elevated levels of SA in *pyr1* plants blunted ET perception. Thus, our results suggested that perception of ABA through PYR1 acted primarily as a module negatively controlling the SA pathway. When ABA/PYR1 failed, the SA pathway was released, and the resistance to *P. syringae* DC3000 was activated. As a trade-off effect, the enhanced accumulation of the SA pathway blocked the ET pathway, the later required for resistance to fungal necrotrophic pathogens.

## 3. Discussion

Despite the demonstrated role of ABA on the final outcome of immune responses, the specific components of the ABA signaling apparatus and the specific mechanisms that exploit ABA to positively and negatively influence immune responses to specific plant-pathogen interactions have remained largely unknown. Here, we showed that SnRK2s kinases were actively engaged in activating resistance towards *P. cucumerina,* whereas the loss-of-function of any of the three individual SnRK2s compromised this resistance. Furthermore, we demonstrated that PYR1 was pivotal and played a positive role in disease resistance to *P. cucumerina* since overexpression of PYR1 (i.e., *PYR1-OE* transgenic line) conferred significantly enhanced resistance. Conversely, in PYR1 loss-of-function mutants, the resistance was compromised. Therefore, the PYR1 receptor had functional specificity in perceiving ABA produced in response to fungal infection to activate plant immunity. This study provided novel information about a specific ABA receptor-mediating specific plant immune responses and pinpointed ABA-activated SnRK2s as cardinal components for plant resistance. This information helps construct a functional classification scheme of the different members of the PYR/PYL receptor family with respect to their downstream signaling pathways in a true biological context. Thus, specific non-redundant roles for PYR1 and PYL8 have been reported in plant immunity (this work) and root ABA sensitivity [50], respectively. An explanation for the specific role of PYR1 in pathogen response could be the selective and highly localized pathogen-induced expression of *PYR1* in vascular bundles (Figure 3A). This expression pattern mirrors the expression of genes encoding ABA-biosynthetic enzymes [65,66,67,68]. Therefore, the synthesis of ABA and the pathogen-induced expression of *PYR1* spatially concur in the vasculature, supporting the hypothesis that vascular tissues function as an integrating node, triggering stress signaling that sets in motion the local and systemic immune responses in the plant [67,69,70].

This study showed that resistance to *A. brassicicola* was also dependent on ABA and PYR1, reinforcing the importance of this signal pathway for activating immunity against necrotrophs. This further reconciled with results shown above and also with previous studies showing that ABA promotes enhanced resistance to the necrotroph *P. cucumerina* [23,25,71]. Moreover, when a fungal necrotroph is a shift to a biotrophic lifestyle by changing the inoculation method and also the developmental stage of the plant [72], as reported for *P. cucumerina* [22], then ABA exerts an opposite effect, and the resistance to this same pathogen is suppressed. This contradictory role of ABA at controlling the disease resistance has also been observed for biotrophic pathogens (e.g., *P. syringae* DC3000), with resistance appearing negatively regulated by ABA, whereas resistance is enhanced in ABA-deficient mutants (Mohr & Cahill, 2007; Jensen et al., 2008; Fan et al., 2009; Verhage et al., 2010) [4,21,26,27]. In fact, we showed that the growth of *P. syringae* DC3000 was severely restricted in *pyr1* plants, as documented for *aba2 aao3* plants or the ABA-insensitive *abi1-1* and *abi2-1* mutants [8,28]. These results demonstrated the Janus functions of PYR1 in disease resistance, mediating repression of immunity against biotrophic pathogens, whereas activation against necrotrophs. Consequently, PYR1 may regulate which of these two plant immune programs prevails. This hypothesis supports previous observations of ABA as a hormone that interacts antagonistically or synergistically with the SA-JA-ET backbone of the plant immune signaling network, redirecting defense outputs [4,28,73,74,75]. Yet, how does the ABA/PYR1 module interfere with immunity to drive simultaneously the repression and activation of the SA and JA/ET defense pathways, respectively? Hormone cross-talk allows different hormone signaling pathways to act antagonistically or synergistically, providing the powerful regulatory potential to flexibly tailor the plant’s adaptive response to a range of environmental cues [4]. Our results showed basal activation of the SA-dependent pathway in *pyr1* mutants, and that *pyr1* was insensitive to the damping effect of ABA on SA signaling. This finding supported previous work demonstrating the negative role of ABA on disease resistance to biotrophs, and that *P. syringae*-induced ABA levels in Col-0 suppress SA biosynthesis and action, enhancing susceptibility to this pathogen [21,28,73,75,76]. Interestingly, our finding that JA perception remained intact in *pyr1* plant but ET perception became compromised added a degree of specificity for the understanding of the disease resistance phenotype of the mutant. The observation that in *pyr1;NahG* plants, the *pyr1*-mediated ET-insensitivity was reversed, and that SA *per se* could block ET perception in Col-0 plants (Figure 8 and Huang et al., 2020), pointed towards SA-mediated repression of ET signaling modulated by ABA and PYR1 during pathogenesis. The positive effect of ABA at promoting ET-dependent resistance to fungal pathogens may be indirect: perception of pathogenic ABA by PYR1 dampens SA signaling, which, in turn, stops ET pathway repression by SA. This ABA and PYR1-modulated cross-talk regulation of SA and ET pathways may provide the plant with a powerful regulatory potential to boost its defenses according to the lifestyle of the attacker. This phenomenon may also explain why disease-promoting biotrophic pathogens (e.g., *P. syringae* DC3000) have developed strategies to alter the host ABA physiology as part of the infection strategy [28,76]. 

How does then ABA/PYR1-mediated signaling control SA-mediated defenses? *pyr1* plants bear constitutive activation of *ICS* expression and moderate enhanced level of SA. Besides, *pyr1* plants carry the hallmarks of immune priming, including (1) basal activation of MPKs; (2) repression of *NRPD2* and, therefore, the RdDM mechanisms that negatively control the onset of defense; (3) activation of the SBT3.3 subtilase; and (4) readying of SA-related genes for enhanced expression by pertinent chromatin modifications. Therefore, *pyr1* plants mirror the phenotypes of RdDM defective mutants, which exhibit simultaneous enhanced susceptibility and resistance to necrotrophs and biotrophs, respectively [54], supporting SA signaling activation in *pyr1* plants. The fact that immune priming and SA-mediated resistance are negatively regulated by the RdDM, and that in *pyr1* plants, the ABA repression of SA pathway is relieved, both observations unveil the importance of ABA/PYR1 as new element participating in an epigenetic mechanism of control of gene expression in plant immunity.

## 4. Materials and Methods

### 4.1. Plants Growth Conditions

Arabidopsis thaliana plants were grown in a growth chamber (19–23 °C, 85% relative humidity, 100 mEm^−2^ s^−1^ fluorescent illumination) on a 10-h-light and 14-hr-dark cycle. All mutants and transgenic plants are in Col-0 background; SnRK2.6-HA/OE and SnRK2.2-HA/OE were previously described [77,78]; snrk2.2 snrk2.3 and snrk2.6 were described in [44,45,51]; the triple mutants pp2ca1-1 hab1-1 abi2-2 and abi2-2 hab1-1 abi1-2 were described in [22]; ocp3-1 and aba2-1 mutants described in [25,53], and the triple pyl4 pyl5 pyl8, pyr1 pyl4 pyl8, and pyr1 pyl4 pyl5 mutants, along with the quadruple pyr1 pyl1 pyl2 pyl4 and single pyl1, pyl4, pyr1-1, pyr1-2 and pyr1-8 mutants were described in [41,45]. Transgenic lines carrying pPYR1::GUS, pPYL1::GUS, pPYL4::GUS were described previously [45]. NahG plants were described previously [64].

### 4.2. Gene Expression Analysis 

Total RNA was extracted from plant tissues using TRIzol (Invitrogen, Waltham, MA, USA) and purified by lithium chloride precipitation. Reverse transcription was done using the RevertAid H Minus First Strand cDNA Synthesis Kit (Fermentas Life Sciences, Waltham, MA, USA). Quantitative PCR (qPCR) was performed using an ABI PRISM 7000 sequence detection system and SYBR-Green (Perkin-Elmer Applied Biosystems, Foster, CA, USA). *ACTIN2* and *ACTIN8* were the reference genes. The primers used for RT-qPCR experiments are provided in Table S1. RT-qPCR analyses were performed at least three times using sets of cDNA samples from independent experiments.

### 4.3. Immunoprecipitation of HA–SnRKs and In Vitro Phosphorylation

HA-tagged SnRK2.2 and SnRK2.6 were immunoprecipitated and used for in vitro kinase assay, as described previously [78].

### 4.4. Chromatin Immunoprecipitation

Chromatin isolation and immunoprecipitation were performed, as described [54,62]. Chip samples, derived from three biological replicates, were amplified in triplicate and measured by quantitative PCR using primers for *SBT3.3*, *PR-1*, *WRKY6*, *WRKY53*, and *Actin2*, as reported [54,62]. All ChIP experiments were performed in three independent biological replicates. The antibodies used for the immunoprecipitation of modified histones from 2 g of leaf material were antiH3K4m3 (#07-473 Millipore) and antiH3K9ac (#07-352 Millipore).

### 4.5. Western Blot

Protein crude extracts were prepared by homogenizing ground frozen leaf material with Tris-buffered saline (TBS) supplemented with 5 mM DTT, protease inhibitor cocktail (Sigma-Aldrich), and protein phosphatase inhibitors (PhosStop, Roche). Protein concentration was measured using Bradford reagent; 25 μg of total protein was separated by SDS-PAGE (12% acrylamide *w/v*) and transferred to nitrocellulose filters. The filter was stained with Ponceau-S after transfer and used as a loading control.

### 4.6. Pathogen Assays

*Pseudomonas syringae* DC3000 was grown for two days, and a culture with O.D. 2 × 10^−4^ was used to infect 5-week-old *Arabidopsis* leaves by infiltration, and the bacterial growth was determined following [54,62]. Twelve samples were used for each data point and represented as the mean ± SD of log c.f.u./cm^2^. For *Plectosphaerella cucumerina* and *Alternaria brassicicola* bioassays, 5-week-old plants were inoculated, as described [23,24], with a suspension of fungal spores of 2.5 × 10^4^, 5 × 10^6^, and 5 × 10^6^ spores/mL, respectively. The challenged plants were maintained at 100% relative humidity. Disease symptoms were evaluated by determining the lesion diameter of at least 100 lesions (25 plants per genotype and four leaves per plant) at 3, 12, and 8 days after inoculation with *P. cucumerina* and *A. brassicicola*, respectively. For pathogen-induced callose deposition analyses, infected leaves were stained at 24, 48, and 72 h.p.i. with aniline blue, and callose deposition quantifications were performed, as described by [53].

### 4.7. Determination of Plant Hormones and Metabolites

ABA, JA, SA levels were determined, as described previously [25,71].

## Supplementary Materials

The following are available online at https://www.mdpi.com/1422-0067/21/16/5852/s1, Figure S1: Disease resistance towards *Alternaria brassicicola* in Col-0, *aba2-1*, *pyr1*, *pyl2* and *pyl4* plants and comparison of Col-0 and *pyr1-2* plants upon inoculation with *Altenaria brassicicola*. Figure S2: Response of Col-0 and *pyr1-2* seedlings to JA. Figure S3: Comparative RT-qPCR analysis for the expression of different JA-responsive and biosynthesis genes in either mocked or *P. cucumerina*-inoculated Col-0 and *pyr1* plants. Table S1: primer sequences.
