# Supplementary material for: The Role of ABA in Plant Immunity is Mediated through the PYR1 Receptor
Source: Int J Mol Sci. 2020 Aug 14;21(16):5852. doi: 10.3390/ijms21165852 (PMC7461614; doi:10.3390/ijms21165852)
Supplement: Supplementary file 1 [file ijms-21-05852-s001.zip › Supplementary Information. Text S1- PRIMERS.pdf]

Text S1. Primer sequences.

Destination	Name Primer Sequence	Sequence 5'-3'
qRT ABI4	ABI4 FW	ACCAAACCCTAACCCATAATAATCC
	ABI4 RV	CGTTGAGCGGAGGAAGTTGAT
qRT PR1	PR1 FW	AAGGGTTCACAACCAGGCAC
	PR1 RV	CACTGCATGGGACCTACGC
qRT PR2	PR2 FW	GGGACGGCTCTCGTGGCTACC
	PR2 RV	CGCGCGTTATCGAAACTCGCGG
qRT PR4	PR4 FW	GCGGCAAGTGTTTAAGGGTGAAG
	PR4 RV	TCCAAATCCAAGCCTCCGTTGC
qRT PR5	PR5 FW	AAATATCTCCAGTATTCACATTC
	PR5 RV	AAGTCTGTGGCCATAACAGCAA
qRT SBT3.3	SBT3.3 FW	AGACCTTTAGCGGTGTGTGTGAGT
	SBT3.3 RV	GCAGCGTTAGTTCTTGAGGCTG
qRT NRPD2	NRPD2 FW	TGCCAGCTTTAAGCAATCAAGGG
	NRPD2 RV	CCCAGTAGTAGCCTCGAACTCTTC
qRT NRPE1	NRPE1 FW	GGCGTTGACTTCATCACGGTTG
	NRPE1 RV	AAAGAAGCACCTGCTGTCTGAG
qRT ACT2/ACT8	ACT2 FW	GGTAACATTGTGCTCAGTGGTGG
	ACT2 RV	AACGACCTTATCTTCATGCTGC
	ACT8 FW	GGTAACATTGTGCTCAGTGGTGG
	ACT8 RV	AACGACCTTATCTTCATGCTGC
