# Supplementary figures and images for: The Role of ABA in Plant Immunity is Mediated through the PYR1 Receptor
Source: Int J Mol Sci. 2020 Aug 14;21(16):5852. doi: 10.3390/ijms21165852 (PMC7461614; doi:10.3390/ijms21165852)

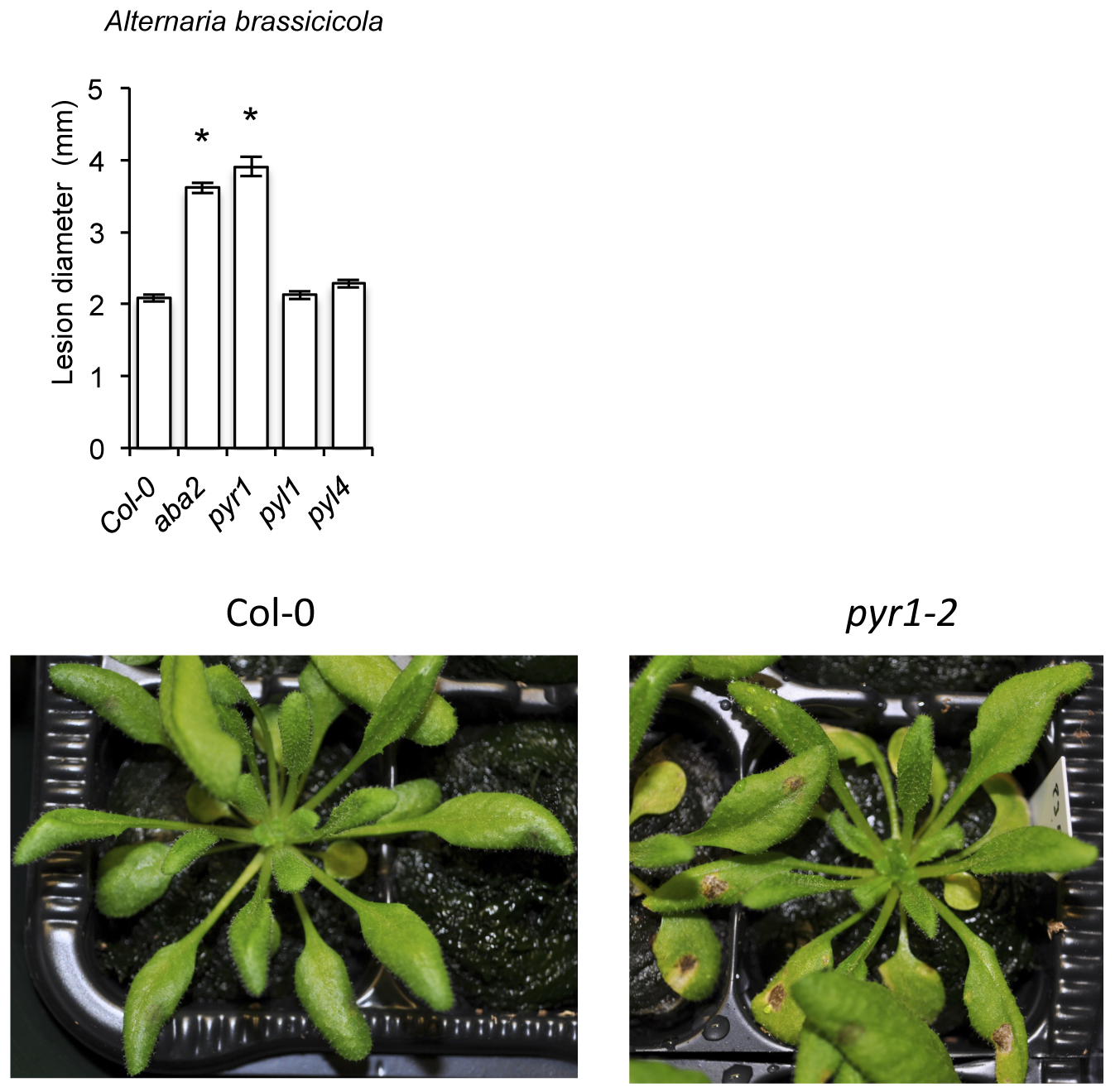

Supplement: Supplementary file 1 [file ijms-21-05852-s001.zip › Supple. Fig 1.tif]

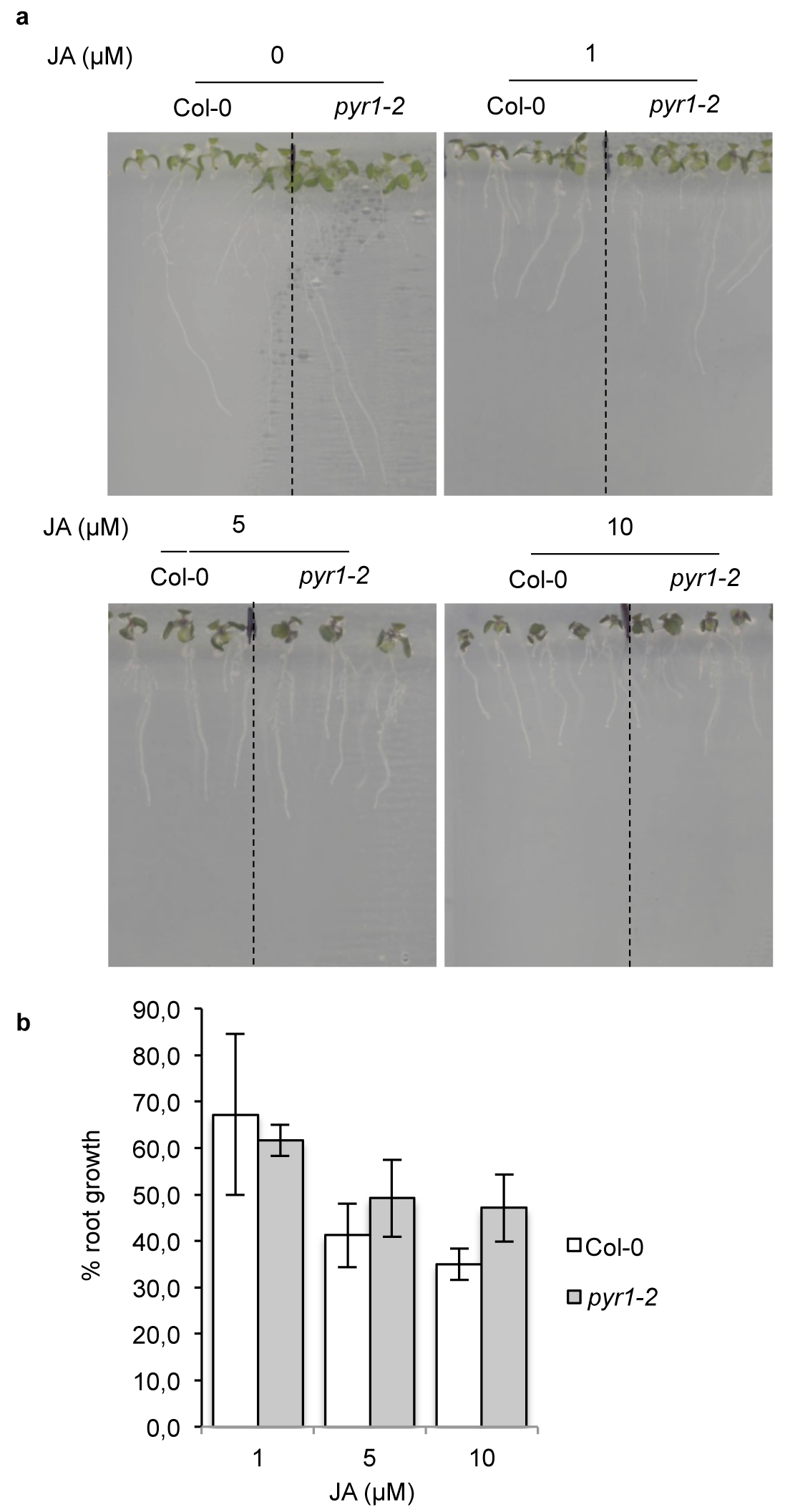

Supplement: Supplementary file 1 [file ijms-21-05852-s001.zip › Supple. Fig 2.tif]

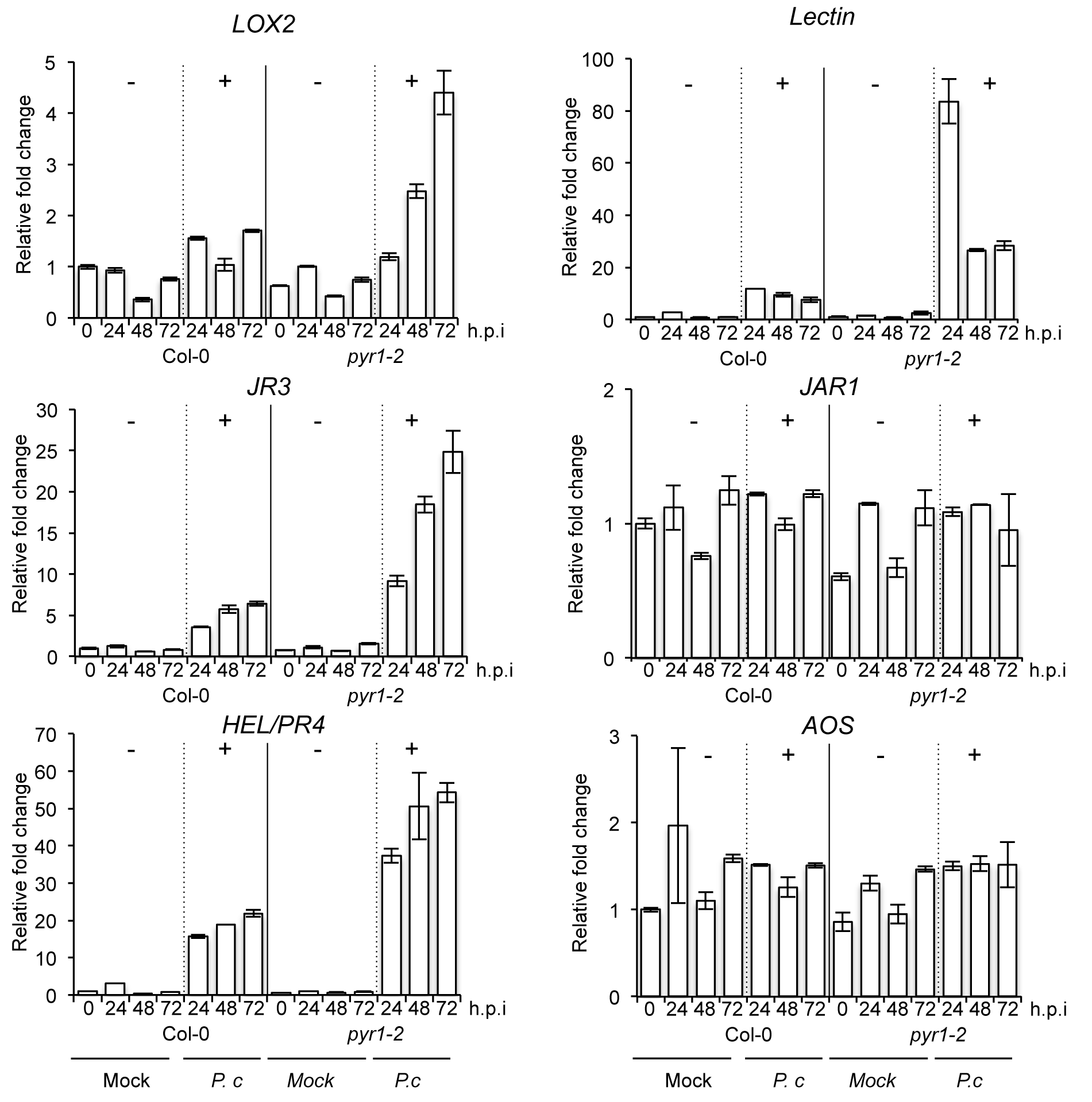

Supplement: Supplementary file 1 [file ijms-21-05852-s001.zip › Supple. Fig. S3.tif]
